# Chronic non-cancer pain management in primary care

**DOI:** 10.4102/safp.v62i1.5203

**Published:** 2020-09-04

**Authors:** Tasleem Ras

**Affiliations:** 1 Division of Family Medicine, Faculty of Health Sciences, University of Cape Town, Cape Town, South Africa

**Keywords:** chronic pain, non-cancer pain, primary care, case study, management of chronic pain

## Abstract

Chronic non-cancer pain is a common, often undiagnosed condition in primary care across the world, with prevalence rates between 20% and 40%. To effectively address this problem, the primary care practitioner needs to have an organised, comprehensive approach to diagnosing and managing these patients within a biopsychosocial framework, in collaboration with members of the interdisciplinary team. The aim of this article is to provide the primary care practitioner with up to date information on the management of chronic pain. A case study is introduced to raise awareness of some of the complexities of dealing with the problem. A series of key questions are raised that address the various levels of complexity. Current evidence is used to guide the reader through these questions, covering a wide area of pain research as it pertains to primary care. The article concludes with five practice points that link the literature to clinical practice.

## Introduction

This article proposes that the clinician needs to answer a series of key pain-related questions to be able to comprehensively care for this patient:

- What is the pain diagnosis?
- Is there an underlying clinical diagnosis?
- What is the impact of the pain?
- What factors (comorbidities) will affect the outcomes?
- What is the best treatment option for this patient?

The summary in Box 1 describes a typical patient presenting to primary care, offering some interesting learning opportunities.

BOX 1 Case study 1.

**Table:** 

Mrs A is a 58-year-old lady who presents for her 6 monthly ‘chronic’ visit. She is known to have reasonably well-controlled hypertension with mild renal impairment. Her last eGFR was 59 g/dL. Her body mass index (BMI) is 39. She complains to the doctor that she has been experiencing severe lower back pain for many years, which has worsened over the last 5 months by radiating down the back of her left leg to her foot. She reports no bladder or bowel functional impairment and no perineal symptoms.
Because of the persistence of the pain, she reports that she is irritable all the time, has trouble sleeping at night and struggles to complete her tasks at work (she works in a textile factory).
After examining her, the doctor makes a clinical diagnosis of left-sided radiculopathy (L4–L5, S1) with associated mechanical back pain, and recognises that her renal dysfunction and obesity will complicate decision-making for therapeutic intervention.

eGFR, estimated glomerular filtration rate.

## The pain diagnosis

Chronic non-cancer pain (CNCP) is a common condition in primary care anywhere in the world, with an estimated prevalence that ranges from 20% to 40%, with a 2013 Pretoria-based study suggesting a 41% prevalence in a South African context.^1^ The International Association for the Study of Pain (IASP) defines chronic pain as pain lasting for more than three months, often accompanied by distress, demoralisation and functional impairment, with significant suffering and economic impact. It is further classified into primary and secondary pain.^2^

When making a pain diagnosis, it is useful for the clinician to decipher if the mechanism of pain is related to underlying tissue damage (nociceptive), a neurological mechanism (neuropathic), a central component (nociplastic) or is a mixture of the preceding three. In the case of Mrs A presented here, a mixed picture of underlying tissue damage emerges (likely spinal degenerative disease), with associated neuropathy and a strong emotional component. This ‘pain diagnosis’ is important as it helps the clinician to make decisions about subsequent pharmacological and non-pharmacological interventions.

Since May 2019, chronic pain has been recognised in the International Classification of Disease , 11th revision (ICD-11) as an independent entity (code – MG30), and a classification system seeks to standardise its reporting.^3^

## The clinical diagnosis

Understanding the clinical diagnosis is central to providing holistic care to this patient. A clinical diagnosis, based on appropriate investigations or interdisciplinary referrals, allows for the implementation of an evidence-based intervention, identifies any reversible cause of the pain and allows a prognosis to be made about disability. However, it must be stated that in many patients, a clear diagnosis is not made.

## Assessing the impact of pain

It is well recognised that any measurement of pain is a subjective process. This is understandable given the complex interaction of physiological pain pathways and the patient’s well-being.

Pain scales focus on pain severity and are useful clinically, but tend to limit the assessment to the physical experience of pain.^4^ Tools that additionally assess the emotional, social and functional impact of chronic pain are more useful in the primary care context.

**FIGURE 1:**
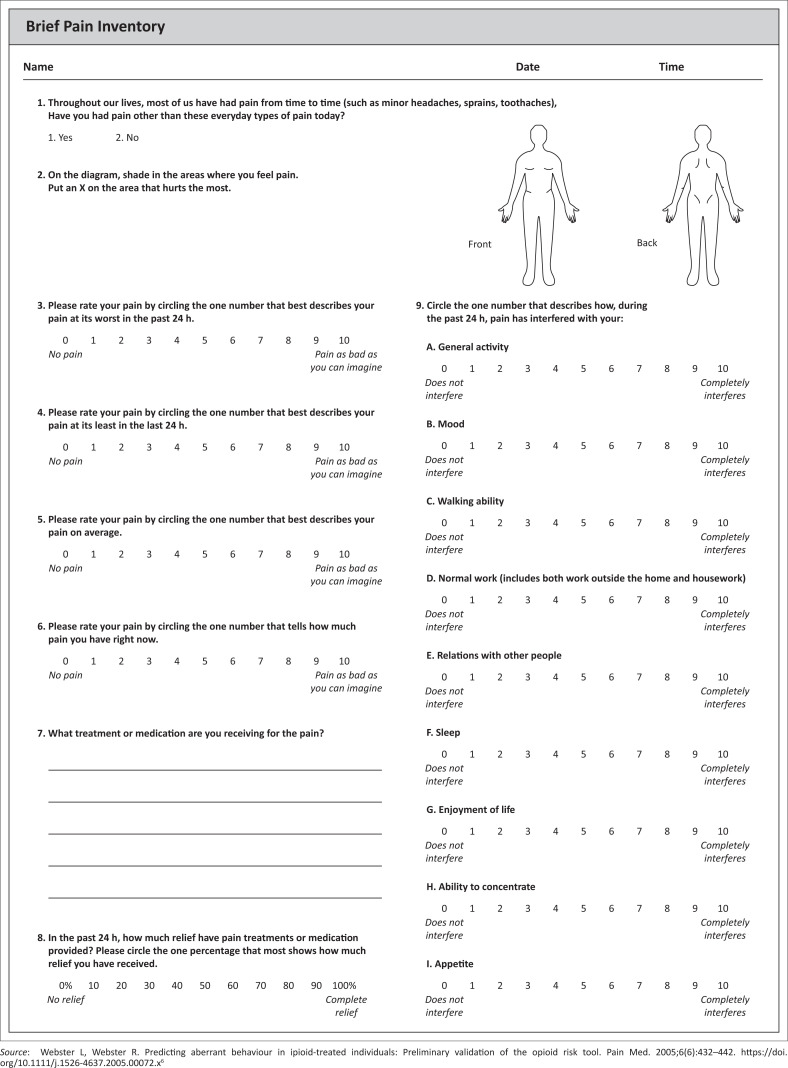
The Brief Pain Inventory.

One such scale is the Wisconsin Brief Pain Inventory (BPI), used widely since its development in 1983, and cross-culturally validated in various contexts.^5^ This tool consists of nine questions (Figure 1) that can be answered by the patient even before the consultation. When used repeatedly, the BPI can be used to track the pain experience over time. The focus on functional ability and quality of life is important, as this becomes a focus when setting treatment goals, given the fact that varying levels of pain will likely persist in the long term.

## Comorbidities that influence pain outcomes

In the above scenario, the obvious comorbidity that impacts this patient’s illness experience is obesity. Obesity has a direct correlation with various types of chronic pain, with increasing BMI associated with increasing pain.^7^

Psychiatric diagnoses, especially the mood and anxiety disorders that impact pain, can be more subtle and need specific enquiry.^8^ Mrs A reports being irritable most of the time, suggesting underlying depression. A comprehensive intervention should include managing any of these emerging diagnoses.

The awareness of opiate addiction has been heightened by the opiate epidemic in North America.^9^ Although there is no African or South African data to describe the situation in our context, the high prevalence of chronic pain necessitates that the primary care physician is aware of this risk and uses an evidence-based tool to guide decision-making. The Opiate Risk Tool (ORT) is a validated, reliable and simple clinical aid that can be administered in a few minutes and stratifies the risk of abuse for an individual patient.^6^

## Evidence-based interventions

Where indicated, non-pharmacological interventions are as, and in some instances more, effective than pharma interventions.

Physical therapy, as administered by physiotherapists primarily, should be part of the first line of therapy for patients suffering from chronic pain relating to musculoskeletal or neuropathic problems. The evidence is very strong to support a range of physical therapy interventions that focus on physical activity, exercise and manual therapy.^10^

Psychological interventions have widespread utility in the long-term management of chronic pain.^11^ Operant conditioning facilitates the engagement of the patient with his or her experience of pain by reconditioning his or her response to the presence of pain. Stress reduction techniques have shown significant effects in managing pain, as it is established that emotional and psychological stress has a direct correlation with pain intensity. Cognitive behavioural therapy seeks to empower the patient to take control of his or her negative thought processes that often accompany chronic pain, which impacts on social and occupational functionality.

Community-based pain groups arranged either as peer support groups or as group interventions led by a professional have been shown to have a significant effect on pain.^12^ These groups have the added dimension of patient education and empowerment, providing patients with validated information that assists them in understanding their illness and facilitates them being part of the therapeutic decision-making process.

Pharmacological interventions should follow the World Health Organisation’s (WHO) pain ladder. The use of fixed dose combinations is generally not recommended.

Paracetamol is still recommended as a first line agent. The clinician needs to educate the patient about dose optimisation, as this is likely the most important reason for patients having a negative attitude to taking this safe medication.

Non-steroidal anti-inflammatory agents (NSAIDs) have a definite place in the management of chronic pain when the underlying pathological process has an inflammatory component.^13^ When used, they should be limited to a few days duration with the intention to suppress the acute inflammation, as long-term use has been associated with poor renal, cardiac and gastro-intestinal (GIT) outcomes. The newer Cox-2 inhibitors have the same precautions attached to them, as the only benefit they offer are a longer half-life (less daily doses required) and slightly lower GIT symptoms, but carry the same risk of renal and cardiac outcomes as the older NSAIDs.

The use of opiates (including the use of the opioid tramadol) has not been shown to be effective when used as a long-term intervention for chronic pain.^14^ Current evidence supports the use of opiates for short-term pain relief only.

The patient in the given scenario has a neuropathic component to her pain. In this instance, amitriptyline would be an appropriate agent to commence treatment with. This tried and tested anti-depressant, when used in doses ranging from 10 mg to 75 mg, has been shown to have an impressive pain modulating impact for neuropathic pain, with a number need to treat (NNT) of 3.6.^15^ The key limiting feature for using amitriptyline is its side effect profile. However, with the low doses used in pain management, this is often not a serious consideration. Escalating doses in excess of 75 mg will not improve pain outcomes. Other medications useful for treating neuropathic pain include the anti-epileptic agents gabapentin (NNT = 6.3 for post-traumatic and trigeminal neuralgia only), pregabalin (NNT = 7.7 for diabetic neuropathy) and carbamazepine (NNT = 1.7 for trigeminal neuralgia only).^15^ Duloxetine, the serotonin-noradrenaline reuptake inhibitor (SNRI) is effective for patients with diabetic neuropathy (NNT = 6.4) and fibromyalgia (NNT = 5–8).^15^

## Conclusion

Box 2 summarises the key learning points for the busy practitioner.

BOX 2 Summary of key practice points.

**Practice points:**

- The effective management of chronic pain is dependent on a strong doctor–patient relationship, as the patient’s engagement is essential to defining treatment goals and monitoring treatment response over time.
- The doctor should see himself or herself as part of a multidisciplinary team that could include physical, psychological and social practitioners.
- Pharmacological interventions should be rational and evidence-based, and non-pharmacological modalities must be a core part of the holistic plan.
- Focussing on improving quality of life and functional ability as treatment goals are more desirable than exclusively focussing on pain severity as the only outcome.
